# Combining independent protein and cellular SELEX with bioinformatic analysis may allow high affinity aptamer hit discovery

**DOI:** 10.1016/j.omtn.2023.06.011

**Published:** 2023-07-18

**Authors:** Coline Ducrot, Max Piffoux

**Affiliations:** 1 Pediatric Orthopedic Surgery Unit, Hôpital Femme Mère Enfant, Hospices civils de Lyon, Bron, France; 2 Team Cell Death and Pediatric Cancer, Cancer Initiation and Tumor Cell Identity Department, INSERM1052, CNRS5286, Cancer Research Center of Lyon, Lyon, France; 3 Medical Oncology, Hospices Civils de Lyon, CITOHL, Lyon, France; 4 Medical Oncology, Centre Léon Bérard, Lyon, France

**Figure 1:**
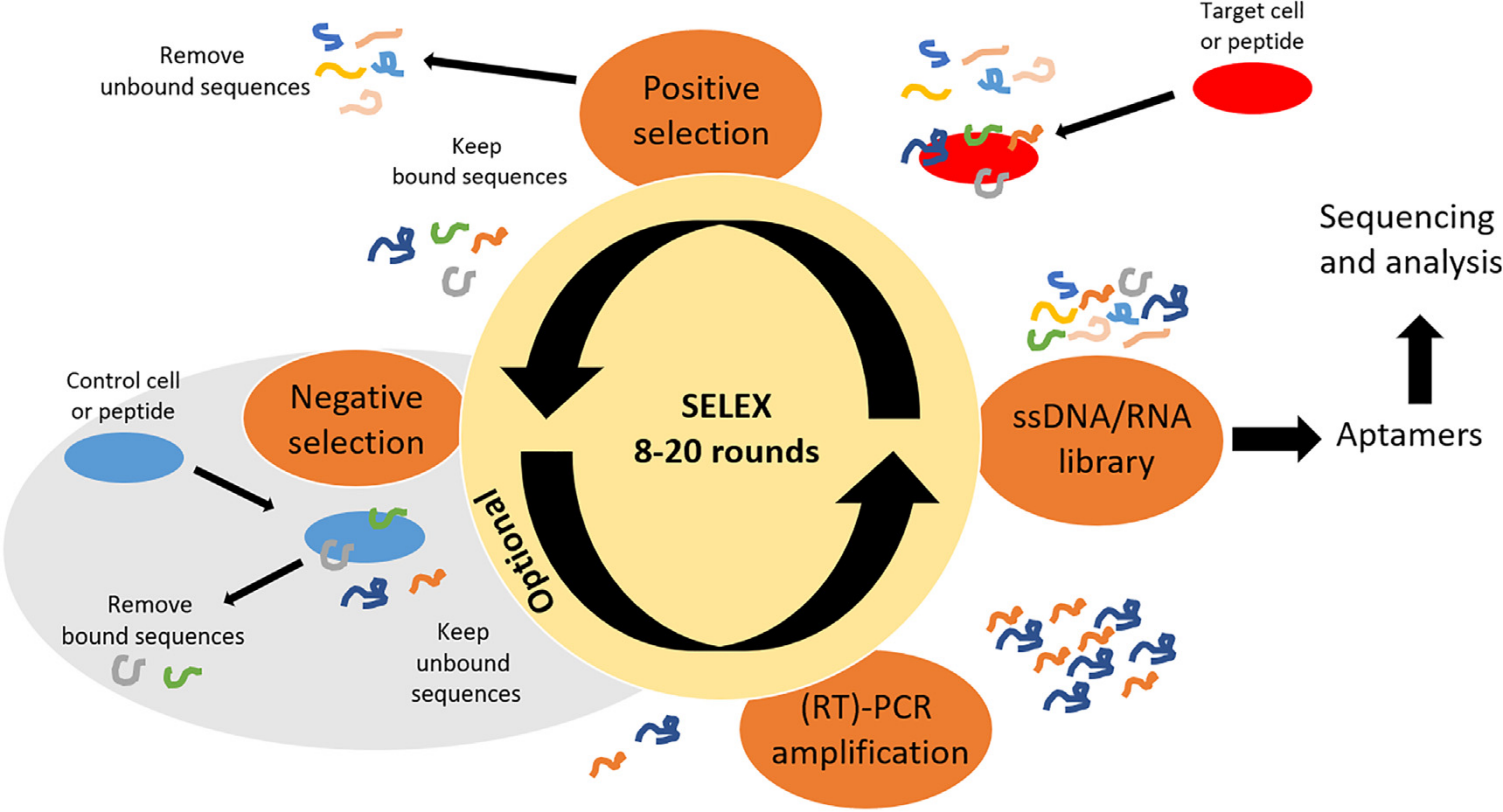
SELEX principle From a single-strand DNA/RNA library, sequences that bind to the target are kept (positive selection). They may optionally be incubated with a target to avoid (negative selection step) and unbound peptides are kept. They are then amplified using RT-PCR to obtain a new and more affine library that may undergo another round of selection or be sequenced and analyzed.

Screening for hits against a target may be performed with various screening methods, like phage display for peptides and antibodies or SELEX (Systematic Evolution of Ligands by EXponential enrichment) for aptamers (Figure 1). Aptamers are small oligonucleotides (typically 20–100 bases) with various 3D structures that may bind to protein targets. Aptamers may be used as diagnostic or therapeutic tools, for example to perform targeted drug delivery or to block a protein function. From a statistical point of view, hit identification may lead to false positives, particularly in case of large libraries. Aptamer libraries have a large diversity, typically 10^15^ (versus 10^11^ for phage display). In this context, identification of aptamers with good properties is rather challenging and usually requires a large number of selection rounds. Screening may be performed against the purified protein target^1^ (Table 1), usually leading to high-affinity aptamers but is at risk of selecting aptamers that bind to an incorrectly folded protein. On the other side, selection may be performed directly on cells,^2^ coupling a positive selection on cells expressing the protein of interest, and a negative selection on cells that do not express the target protein. This typically leads to selection of lower affinity aptamers but specific to the normally folded protein target.

**Table 1:** SELEX methods

	Basal techniques		Complex/combined techniques			
	Protein SELEX	Cell SELEX	Ligand guided SELEX	Toggle SELEX	Hybrid SELEX	Independent and combined SELEX
Concept	Use a purified protein target for selection	Use a cell expressing the protein of interest for positive selection, needs to be coupled to a negative selection with similar cells that do not express the protein of interest	Use a ligand (e.g., antibody) with high affinity directed against an epitope of the target protein during positive selection to remove aptamers specific of this epitope	Alternating targets (e.g., two protein isoforms) or methods (e.g., cell and protein SELEX) at each round	Performing cell and protein SELEX one after another	Performing cell and protein SELEX independently and select aptamers with secondary structure that are shared and enriched in both cases
Ref.	Tuerk and Gold^1^	Morris et al.^2^	Zumrut et al.^3^	White et al.^4^	Uemachi et al.^5^	Santana-Viera et al.^6^
Interest	• Cheap • Fast • High affinity	• Target the *in vivo* tertiary structure of the protein	• Select aptamers with epitope specificity	• Select cross reactive aptamers	• Select aptamers with good affinity and cell internalization property	• Select aptamers with good affinity and cell internalization property • May select fewer false positive hits
Limit	Potentially nonspecific aptamer (cross reactive to other proteins) • May not target the *in vivo* tertiary structure of the protein	• Complex • Low affinity	• Low affinity • Needs to be performed on a pre-enriched aptamer bank	• Complex • Lower affinity	• Complex • Not cross reactive	• Complex • Not cross reactive • Time-consuming

In the June issue of MTNA, Santana-Viera et al.^6^ propose to combine these two selection methods by performing each of them independently and then use a new bioinformatic tool to select conserved and structurally similar sequences. Other authors proposed to combine cellular and protein SELEX, either by changing the method at each round, called “Toggle” SELEX(4), or first performing several rounds of protein SELEX and then several rounds of cellular SELEX, also called “Hybrid” SELEX(5) (Table 1). The authors’ approach led to the identification of a 10^−10^ M affinity aptamer targeting EphA2, a receptor commonly overexpressed in several types of cancer.^7^ This aptamer has an *in vivo* binding capability in a mice model of Ewing sarcoma expressing high levels of EphA2 as well as interesting intrinsic antiproliferative properties, even if not drug loaded. The “independent and combined” SELEX allowed discovery of a good affinity hit, making it a potentially interesting method. It is also expected to be reproducible as it is based on independent experiments. Identification of a candidate EphA2 targeting aptamer may be of interest in Ewing sarcoma but also in many other EphA2 expressing cancers.

Compared with antibodies, aptamers are produced by chemical synthesis, a process that is industrially more reliable, less expensive, and less variable than cell-based production. Aptamers are also expected to be less prone to the development of anti-drug antibodies and have better tumor-penetrating capabilities due to their small size (20 kDa versus 150 kDa^8^). On the other side, aptamers do not benefit from Antibody Dependent Cytotoxicity (ADCC) or antibody’s interesting long half-lives. Aptamers are usually produced using modified nucleotides that resist blood nucleases and typically have a short half-life of 5–15 h that may be increased to 2–4 days using PEGylation. The short half-life of aptamers may potentially be of interest in some indications.

EphA2 is a tyrosine kinase receptor whose expression level is linked with tumor aggressiveness. Once bound to its ligand ephrin, it activates a canonical signaling pathway that negatively regulates cell proliferation and induces EphA2 degradation. Ephrin is a membrane protein that activates EphA2 when cells are in close contact. Cancer cells tend to have a looser cell-to-cell contact, leading to EphA2 overexpression. When not bound to ephrin, EphA2 also has an autonomous activation that switches on a non-canonical signaling pathway leading to an increased cell invasiveness and proliferation. EphA2 is therefore an interesting target as it is both a cancer overexpressed target and its targeting induces an anti-proliferative effect even in the absence of combination with a cytotoxic payload.

In their article, Santana-Viera et al. identified a 2′-fluoro-modified RNA aptamer that reduced cell proliferation and migration *in vitro*, as well as *in vivo* where it reduced primary tumor and metastasis growth in a xenografted Ewing sarcoma murine model.

Their aptamer was the top candidate obtained using parallel and independent protein and cellular SELEX whose results were analyzed together using a new bioinformatic pipeline. Briefly, aptamer’s secondary structure is predicted and a clustering algorithm grouped them based on their structural similarity. The protein SELEX aptamer that exhibited the highest number of connections with the cellular SELEX was identified, and its neighbors most common sequences/motifs were used to design the final candidate aptamer. The proposed aptamer has higher affinity than previously published ones targeting EphA2.

The proposed method couples the interest of both protein and cellular SELEX, just like the « toggle » and « hybrid » SELEX methods (Table 1). From a statistical point of view, the two experiments are in that case performed independently, probably leading to fewer false positive results. On another side, this study is only a proof of concept on a single case, and real head-to-head comparisons of SELEX methods are required to better understand the relative interest of each method and optimize parameter selection in the bioinformatic pipeline. The authors’ concept of dual independent screening combined with the use of structural or sequence similarity analysis may also be of interest for other screening methods like phage display.

The authors chose to demonstrate their aptamer *in vivo* properties in a xenografted murine model of human Ewing sarcoma, implying that the human EphA2 target is only expressed in the tumor. The relatively high tumor-specific targeting ability observed may be substantially lower in humans where EphA2 is also expressed at a basal level in many epitheliums. Toxicity evaluation, especially if loaded with cytotoxic drugs, will in this context require further evaluation. Other EphA2 targeting drugs or CAR T cells are in preclinical or clinical development (phase I trials) but usually with limited, unpublished, or negative results.^7^

Only one aptamer (Pegaptanib) has been approved by the Food and Drug Administration since 2004 as the first anti-VEGF agent used in neovascular age-related macular degeneration.^9^ It has since been replaced by anti-VEGF antibodies (Lucentis and Avastin) that have shown better clinical efficacy. This may interestingly be explained by the fact that Pegafitinib specifically targets one isoform of VEGF, whereas anti-VEGF antibodies are cross-reactive to all isoforms. Interestingly, this aptamer was PEGylated to achieve longer half-life. PEGylation may also be of interest for EphA2 targeting aptamers in the future, as well conjugation with cytotoxic drugs or antisense oligonucleotides.
